# Temperate phages enhance pathogen fitness in chronic lung infection

**DOI:** 10.1038/ismej.2016.51

**Published:** 2016-04-12

**Authors:** Emily V Davies, Chloe E James, Irena Kukavica-Ibrulj, Roger C Levesque, Michael A Brockhurst, Craig Winstanley

**Affiliations:** 1 Department of Clinical Infection, Microbiology and Immunology, Institute of Infection and Global Health, University of Liverpool, Liverpool, UK; 2 School of Environment and Life Sciences, University of Salford, Manchester, UK; 3 Institut de Biologie Intégrative et des Systèmes (IBIS), Université Laval, Québec, Québec, Canada; 4 Department of Biology, University of York, York, UK

## Abstract

The Liverpool Epidemic Strain (LES) is a polylysogenic, transmissible strain of *Pseudomonas aeruginosa*, capable of superinfecting existing *P. aeruginosa* respiratory infections in individuals with cystic fibrosis (CF). The LES phages are highly active in the CF lung and may have a role in the competitiveness of the LES *in vivo*. In this study, we tested this by competing isogenic PAO1 strains that differed only by the presence or absence of LES prophages in a rat model of chronic lung infection. Lysogens invaded phage-susceptible populations, both in head-to-head competition and when invading from rare, in the spatially structured, heterogeneous lung environment. Appreciable densities of free phages in lung tissue confirmed active phage lysis *in vivo*. Moreover, we observed lysogenic conversion of the phage-susceptible competitor. These results suggest that temperate phages may have an important role in the competitiveness of the LES in chronic lung infection by acting as anti-competitor weapons.

The LES is a transmissible strain of *Pseudomonas aeruginosa* that causes life-limiting chronic respiratory infections in individuals with CF (Fothergill *et al.*, 2012). Unusually, the LES is capable of superinfection to displace other strains of *P. aeruginosa*, even after years of chronic colonisation (McCallum *et al.*, 2001; Winstanley *et al.*, 2009), but there is no evidence that it can be displaced by other strains (Fothergill *et al.*, 2010; Mowat *et al.*, 2011; Williams *et al.*, 2015). The LES harbours five prophages, highly active in the CF lung (James *et al.*, 2015), four of which have been shown by signature-tagged mutagenesis to be necessary for bacterial competitiveness in a rat model of chronic lung infection (Winstanley *et al.*, 2009; Lemieux *et al.*, 2015). Theoretical and empirical studies suggest that temperate phages could enhance the competitiveness of lysogens by killing phage-susceptible competitors by lysis (Bossi *et al.*, 2003; Brown *et al.*, 2006; Joo *et al.*, 2006; Burns *et al.*, 2014), a form of phage-mediated allelopathy (Stewart and Levin, 1984). However, it is unclear whether this ecological mechanism operates in the far more complex spatially structured and heterogeneous lung environment.

To test whether temperate phages increase competitive fitness during lung infection, we performed competition experiments between lysogenic and non-lysogenic strains of *P. aeruginosa* in a rat model of chronic lung infection (Winstanley *et al.*, 2009). We constructed antibiotic-resistance-labelled PAO1 LES-Phage Lysogens (PLPLs) (James *et al.*, 2012) using three of the LES phages (LESφ2, LESφ3 and LESφ4), both individually and in combination (for full methods, see Supplementary information). Initial *in vitro* experiments suggested that the triple lysogen (PAO1φtriple) was more invasive than any of the constituent single lysogens (Supplementary Figure S1), therefore PAO1φtriple was selected for use in the *in vivo* experiments. Competitors were embedded in agar beads in PLPL to PAO1^φ−^ ratios of 1:5 or 1:1, to model invasion-from-rare and head-to-head competition, respectively. Rats were infected with inoculated agar beads by intubation and monitored for 7 days, after which they were killed. The densities of each competitor in the lungs were quantified (Supplementary Table S2) and the selection rate constant (*r*_ij_) was calculated as described previously (Lenski *et al.*, 1991).

PAO1φtriple outcompeted PAO1^φ−^ *in vivo* (Figure 1), at both initial starting frequencies (competition experiment, 1 sample *t*-test (alt=0), *t*_6_=20.3, *P*<0.001; invasion experiment, 1 sample *t*-test (alt=0), *t*_3_=15.8, *P*<0.01), but there was no effect of initial starting frequency on fitness (2-sample *t*-test; *t*_6_=1.08, *P*=0.32). Thus, temperate phages improved the competitiveness and invasiveness of lysogens against phage-susceptible populations in chronic lung infection. To confirm the role of phage lysis, we also measured levels of free infective phages in the lung homogenate. We observed appreciable densities of virions in the lungs in both treatments, with no significant difference in mean (±1 s.d.) phage-to-bacterium ratios between the competition (0.55±0.84) and invasion (0.47±0.44) treatments (2-sample *t*-test on log_10_+1 transformed data; *t*_6_=−0.11, *P*=0.91).

Phage-mediated invasion by lysogens can be limited by lysogenic conversion of the originally phage-susceptible competitor, creating phage-resistant lysogens (Gama *et al.*, 2013). Having found that lysogenic conversion occurs at a very high frequency *in vitro* (Supplementary Figure S2), we investigated whether the phages also established lysogeny *in vivo*. We calculated for each animal the total lysogen frequency and proportion of each lysogen type for end point populations by screening 46 bacterial lung isolates of the initially phage-free competitor (PAO1^φ−^) using a multiplex PCR assay (Supplementary Methods and Supplementary Table S1). We observed appreciable rates of lysogenic conversion at both initial starting frequencies, but with substantial variation between animals (Figure 2). Although the mean (±1 s.d.) total frequency of lysogens was higher in head-to-head competition (0.89±0.03) compared with invasion-from-rare (0.56±0.07) treatment (2-sample *t*-test; *t*_6_=8.57, *P*<0.01) (due to a proportion of the PLPLφtriple competitor), the rate of lysogenic conversion of PAO1^φ−^ (Figure 2) was similar in both treatments (Mann–Whitney *U* test; *W*=42.5, *n*_1_=7, *n*_2_=4, *P*=1.00). Lysogenic conversion of PAO1^φ−^ was dominated by the formation of LESφ2 and LESφ3 lysogens, suggesting that these phages were most active in the lung, which is consistent with the high free-phage densities of these phages in human CF infections (James *et al.*, 2015).

These data provide important experimental evidence supporting the role for phage-mediated allelopathy as a determinant of pathogen fitness in chronic lung infection. This extends previous studies using observational (James *et al.*, 2015) and insect model approaches (Burns *et al.*, 2014) to confirm, in a clinically relevant environment, that the LES-temperate phages are likely to have had a key role in the global spread of the LES. Crucially, we demonstrate that phage-mediated allelopathy allows lysogens to invade from rare, even in the complex, spatially structured, heterogeneous host lung environment, which has previously been theoretically predicted, but has never been demonstrated (Gama *et al.*, 2013). In agreement with a recent observational clinical study of the ecological dynamics of the LES and its phages in CF patient sputa, we show the production of appreciable populations of free-phage virions by lysis in the lung (James *et al.*, 2015). We observed lysogenic conversion in the lung, but at rates lower than those observed in liquid *in vitro* environments (Supplementary Figure S2), suggesting that lysogenic conversion may have been impeded in the lung environment. Consistent with this, recent evidence suggests that bacterial populations show strong regional structure within the CF lung, with low rates of mixing between regions of the lung (Jorth *et al.*, 2015). Nevertheless, the transfer of genetic material among strains of *P. aeruginosa* within infections does raise concerns about the potential for the horizontal gene transfer of antibiotic resistance or virulence determinants (Penadés *et al.*, 2015).

## Figures and Tables

**Figure 1:**
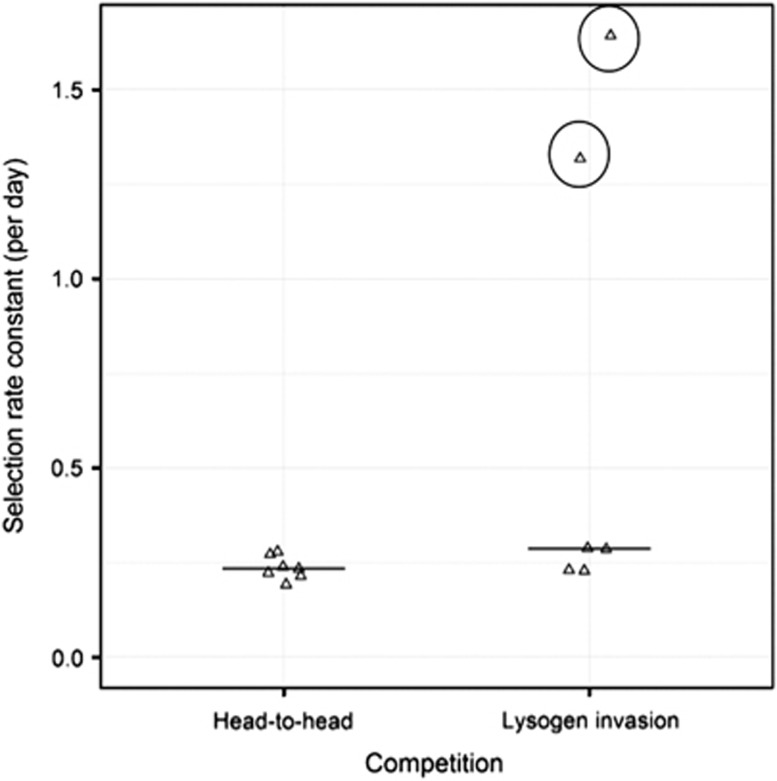
Selection rate constant for the competition outcome between PAO1^φ−^ and PAO1φtriple at different starting ratios of competitors. Each data point represents the outcome of competition in the lungs of an individual animal after 7 days, with the exception of the two circled data points. These represent two rats that were killed after 2 days, as they were showing symptoms of acute infection, with high bacterial loads (100-fold higher than other lungs after 7 days). These were excluded from statistical analyses.

**Figure 2:**
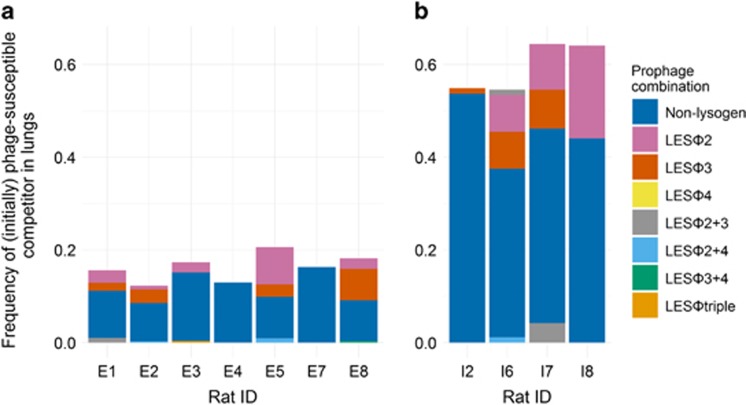
Lysogenic conversion of PAO1^φ−^ after 7 days *in vivo*. Prophage complement of streptomycin-labelled bacteria (initially PAO1^φ−^) isolated from rat lungs in (**a**) head-to-head competition and (**b**) invasion-from-rare treatments. Height of bars denote the frequency of the competitor out of total bacteria. Data are reported separately for each animal.
