# Supplementary material for: Temperate phages enhance pathogen fitness in chronic lung infection
Source: ISME J. 2016 Apr 12;10(10):2553–5. doi: 10.1038/ismej.2016.51 (PMC4950967; doi:10.1038/ismej.2016.51)
Supplement: Supplementary Information [file ismej201651x1.doc]

**Supplementary Methods**

*In vitro invasion assays*

We constructed marked PAO1 LES Phage Lysogens (PLPLs) (James et al 2012) using three of the LES phages (LESφ2, LESφ3 and LESφ4), both singularly and in combination. Competitors were labelled with fitness-neutral fluorescence markers and competitions set up with an initial starting ratio of PLPL to PAO1^φ-^ of 1:9. Each construct was labelled separately with a mini-Tn*7* transposon marker expressing green fluorescent or DsRed-Express protein (Koch et al 2001). Reverse markers were used for each competition to control for any effect of marker.

Both competitors were grown to mid-exponential phase (OD_600_ 0.5) in Luria Broth. Cultures were centrifuged at 12,000 *g* for 2 min, the supernatant discarded and the pellet resuspended in 10 times the initial volume of prewarmed M9-CaGlu. PAO1^φ-^-GFP and PLPL-DsRed-Express were added to a well of a 96-well black polystyrene microtitre plate (Corning, Corning, NY) in the correct ratio, to a final volume of 200 µl, and the mixture pipetted up and down to mix well. This was repeated in another well for the reverse markers, with 6 technical replicates for each mixture. The plate was incubated at 37˚C with shaking at 200 r.p.m. in a Fluostar Omega plate reader (BMG Labtech, Durham, NC). Fluorescence intensity (FI) of both GFP and DsRed-Express in each well was measured every 8 h using 485 nm and 544 nm excitation filters and 520 nm and 590 nm emission filters, for GFP and DsRed-Express, respectively. Readings were normalised to an unlabelled PAO1 negative control to control for bacterial autofluorescence. To allow non-destructive sampling of bacterial densities over the 24 h period, densities were estimated by comparing FI of each well to a standard curve of FI against bacterial density. To produce the standard curve, PAO1 GFP and PAO1 RFP were mixed together, with different ratios in each well (i.e. 10 µl GFP/ 190 µl DsRed-Express, 20 µl GFP/ 180 µl DsRedExpress etc.). Standards were included on every plate. At each time point, the FI for each set of filters was plotted against the bacterial cell densities (predetermined for each time point by plating for c.f.u.), and the line of best fit was calculated using Sigma plot.

Three independent biological replicates were performed for each competition. The Malthusian parameters of each competitor and the selection rate constant were calculated separately for each marker in each competition, at t_24_ only.

Natural log-transformed data were analysed with a repeated measures ANOVA design by fitting a general linear model (GLM). Time and PLPL were fitted as fixed factors, fluorescent marker as a random effect, and competition ID as a random effect, nested within PLPL to account for the repeated measures.

For each of the lysogens used, we confirmed that the relative growth rates did not differ from PAO1 in either Luria broth or an artificial sputum medium.

*In vivo assay*

To distinguish from animal microflora and each other, PAO1φ- and PAO1Φtriple were labelled with streptomycin and gentamicin resistance markers, respectively (Burns et al 2014). It has been shown previously that there is no difference in fitness between PAO1 strains labelled with these two markers (Burns et al 2014). Competitors were grown to an OD_600_ of 1.0. For the invasion assay, 2 x 10^9^ cells PAO1φtriple Gm^R^ and 1.8 x 10^10^ cells PAO1^φ-^ Sm^R^ were centrifuged at 7,200 r.p.m. for 3 min, and washed 3 times in sterile phosphate-buffered saline (PBS). The cell suspensions were resuspended in 500 µl PBS, pooled together and mixed thoroughly. The bacterial suspension was added to 10 ml molten 2% (w/v) agar-PBS, preheated to 48 ˚C, and vortexed vigorously. The mixture was poured slowly into the centre of an Erlenmeyer flask containing 200 ml sterile mineral oil (Sigma-Aldrich, Canada) held at 48 ˚C, with constant magnetic stirring. Ice was added to the sides of the flask to cool, and the stirring continued for 5 min. The stirring was ceased and the flask left at room temperature for 10 min to allow the beads to settle at the bottom of the flask.

The mineral oil was siphoned off using a vacuum pump, and the beads washed with an equal volume of PBS, followed by centrifugation at 10,000 r.p.m at 4 ˚C for 20 min. Residual mineral oil was removed, and this wash-step repeated a further two times to remove all the mineral oil. All but 50 ml of the PBS was removed, and the remaining PBS-bead suspension transferred into a sterile falcon tube and allowed to settle for 10 minutes. The majority of the PBS was removed, to leave approximately 7.5 ml of beads, suspended in an equal volume of PBS. This process was repeated for the head-to-head competition, but using 1 x 10^10^ cells of each competitor. The beads were stored at 4 ˚C prior to infection. For accurate quantification of bacterial densities of each competitor in the beads, 1 ml of the bead suspension was diluted into 9 ml PBS and homogenised (on ice) for 30 seconds, using a PTA 20S Polytron homogeniser (Kinematica AG, Luzern, Switzerland). Bacterial counts were determined by plating onto TSA containing 300 µg ml^-1^ streptomycin or 10 µg ml^-1^ gentamicin, in addition to TSA containing no antibiotic.

Although, the initial aim was for a 1:9 ratio of PLPL to PAO1^φ-^, the loss of bacteria during the bead making process changed the actual ratio of competitors to 1:5. It is worth noting that the competing bacteria would be in contact with each other during the bead preparation process. However, all procedures were carried out on ice to minimize the possibility of competition between strains prior to introduction into the animal.

Ethical approval for animal experiments was obtained from the Animal Care Committee, Laval University. Male Sprague-Dawley rats, weighing between 300-350 g, were housed in individual cages, with food and water *ad libitum*. Rats were weighed, and anaesthetised with 2% (v/v) isofluorane-oxygen, followed by an intra-peritoneal ketamine/xylazine (80/100 mg/ml at 100 µl/100g) injection to maintain anaesthesia. Rats were intubated using an 18-gauge venous catheter, and a 1 ml tuberculin syringe used to deliver between 2 x 10^7^ and 4 x 10^7^c.f.u. into the lungs (in a volume of between 100 and 120 µl). Rats were sacrificed after 10 days, or earlier if showing signs of morbidity using Euthanyl (Bimeda-MTC, Ontario, Canada) administered by intra-peritoneal injection, at a dosage of 240 mg/kg of body weight. Lungs were removed and suspended in 10 ml sterile PBS, on ice. Lungs were homogenised and the c.f.u. in the lungs determined by plating onto antibiotic plates as described for the beads. Selection rate constants were calculated as described above, using actual cell densities in the bead inoculum and lungs as start and end densities, respectively. To quantify free infective phages present in the lung tissue, the lung homogenate was centrifuged and the supernatant filtered using a 0.2 µm filter (Millipore, Milford, MA, USA). Phages in the supernatant were quantified immediately by plaque assay as described in (James et al 2012). The phage to bacterium ratio was calculated as the total number of p.f.u. in the lungs, divided by the total number of c.f.u.

*Multiplex PCR detection of lysogens*

Isolated bacterial colonies were screened for the presence of prophage using a multiplex PCR assay, with primers targeted to each of the LES phages. A single colony was suspended in 50 µl dsH2O and heated to 100 °C for 5 minutes, and 5 µl of the boil preparation was used as a template in the PCR reaction, in combination with 10 pmol each primer, 1.5 mM MgCl_2_, 10 µM each dNTP, 5X GoTaqBuffer and 1U GoTaq polymerase (Promega). Cycling conditions were as follows: 95°C, 4 min then 30 cycles: 95°C, 30 s; 58°C, 30 s; 72°C, 30 s; final extension step, 72°C, 7 min. Primer sequences are detailed in Table S1.

Table S1. Primers used in study

| **Primer name** | **Sequence** | **Target** | **Length of amplicon (bp)** |
| --- | --- | --- | --- |
| LESp2F | ctccacttctcggttgcttc | LES φ2 | 206 |
| LESp2R | actagccccgtatccgagtt |
| LESp3F | tcaggaaaaccttgccattc | LES φ3 | 384 |
| LESp3R | gtcttctggtggtcggtgat |
| Lesp4F | agttacgcctgctggtgagt | LES φ4 | 506 |
| Lesp4R | cctcagtcgtgccttctttc |

**Supplementary Results**

***In vitro* experiments**

All the PLPLs could invade a population of phage-susceptible PAO1^φ-^ from when initially rare (Fig. S1), but there were differences in invasion dynamics depending on prophage carried. There was no effect of marker so it was removed from the model. The main effects of PLPL (*F*_3,72_ = 4.40, *P* < 0.05) and time (*F*_3,72_ = 198.5, *P*< 0.001) were significant, as was the interaction (*F*_9,72_ = 20.1, *P* < 0.001). Post-hoc Tukey HSD tests were performed for the interaction effect, comparing all PLPLs to each other at each time point using the hierarchical model. After 24 h, PLPLφ2 and PLPLφ3 were not as invasive as PLPLφ4 or PAO1Φtriple, suggesting that carriage of LESφ4 is more beneficial to bacteria in this respect than carriage of LESφ2 or LESφ3. Analysis of endpoint isolates detected a high level of lysogenic conversion (Fig S2) which may explain the plateau for LESφ2 and LESφ3, as lysogens become resistant to lytic infection by superinfection exclusion and the allelopathic action of the phage is no longer effective (Burns et al 2014, Gama et al 2013).

Carriage of all three prophages is associated with a more rapid invasion, as PAO1Φtriple is significantly more invasive than all single lysogens after 16 h, potentially because carriage of multiple prophages delays lysogenic conversion of susceptibles.

*In vivo* bacterial densities

Total bacterial density in the bead inocula administered to each rat was 1.6 x 10^7^ c.fu. for the head-to-head and 3.6 x 10^7^ c.f.u. for the invasion from rare treatment. Total bacterial density in the lungs ranged from 2.2 x 10^5^ to 8.1 x 10^6^ c.f.u. (head-to-head) and 1.3 x 10^6^ to 3.9 x 10^7^ c.f.u. (invasion from rare) for rats sacrificed at 7 days. The bacterial densities in the lungs of the two rats euthanised after 2 days ranged from 6.7 to 8.2 x 10^8^ c.f.u. (Table S2).

**Table S2. Bacterial densities at the start and end of the in vivo experiment**

| **Treatment** | **Rat ID** | **Total bacterial density in inoculum (c.f.u.)** | **Total bacterial density recovered from lungs at time of sacrifice (c.f.u.)** | **Length of infection (days)** | **Ratio of competitors (initial lysogen: initial non-lysogen)** | |
| --- | --- | --- | --- | --- | --- | --- |
|  |  |  |  |  | **Start** | **End** |
| **Head to head** | **E1** | 1.55E+07 | 9.6E+05 | **7** | 1 | 5.4 |
| **Head to head** | **E2** | 1.55E+07 | 2.3E+06 | **7** | 1 | 7.1 |
| **Head to head** | **E3** | 1.55E+07 | 8.1E+06 | **7** | 1 | 4.8 |
| **Head to head** | **E4** | 1.55E+07 | 2.9E+06 | **7** | 1 | 6.7 |
| **Head to head** | **E6** | 1.55E+07 | 5.1E+05 | **7** | 1 | 3.9 |
| **Head to head** | **E7** | 1.55E+07 | 3.1E+05 | **7** | 1 | 5.2 |
| **Head to head** | **E8** | 1.55E+07 | 2.2E+05 | **7** | 1 | 4.5 |
| **Invasion from rare** | **I1** | 3.60E+07 | 6.7E+08 | **2** | 0.16 | 1.6 |
| **Invasion from rare** | **I2** | 3.60E+07 | 9.0E+06 | **7** | 0.16 | 0.8 |
| **Invasion from rare** | **I3** | 3.60E+07 | 8.2E+08 | **2** | 0.16 | 3.0 |
| **Invasion from rare** | **I6** | 3.60E+07 | 1.3E+06 | **7** | 0.16 | 0.8 |
| **Invasion from rare** | **I7** | 3.60E+07 | 3.9E+07 | **7** | 0.16 | 0.6 |
| **Invasion from rare** | **I8** | 3.60E+07 | 8.4E+06 | **7** | 0.16 | 0.6 |

For the ratio of competitors, initial lysogen or initial non-lysogen refers to the genetic background at the start of the experiment. The initial lysogens were marked with gentamycin resistance whereas the initial non-lysogens were marked with streptomycin resistance.

**Supplementary figure legends**

Figure S1. Ratio of PLPL to PAO1^φ-^ over 24 hour coculture, with a starting ratio of 1:9 PLPL to PAO1^φ-^. Error bars ±1 S.D. Three biological replicates were performed for each PLPL.

Figure S2. Frequency of bacterial lysogens after 24 h of co-culture of various PLPL with PAO1^φ-^ with an initial starting ratio of 1:9. Error bars ±1 S.D. Three biological replicates were performed for each strain, and a minimum of 46 separate colonies were tested per replicate. For the triple phage experiment, a lysogen is counted as any isolate containing at least one of the phage.

**Supplementary references**

Burns N, James CE, Harrison E (2014). Polylysogeny magnifies competitiveness of a bacterial pathogen in vivo. *Evolutionary applications*.

Gama JA, Reis AM, Domingues I, Mendes-Soares H, Matos AM, Dionisio F (2013). Temperate bacterial viruses as double-edged swords in bacterial warfare. *PloS one* **8:** e59043.

James C, Fothergill J, Kalwij H, Hall A, Cottell J, Brockhurst M *et al* (2012). Differential infection properties of three inducible prophages from an epidemic strain of Pseudomonas aeruginosa. *BMC Microbiology* **12:** 216.

Koch B, Jensen LE, Nybroe O (2001). A panel of Tn7-based vectors for insertion of the gfp marker gene or for delivery of cloned DNA into Gram-negative bacteria at a neutral chromosomal site. *Journal of Microbiological Methods* **45:** 187-195.
