# Supplementary figures and images for: Temperate phages enhance pathogen fitness in chronic lung infection
Source: ISME J. 2016 Apr 12;10(10):2553–5. doi: 10.1038/ismej.2016.51 (PMC4950967; doi:10.1038/ismej.2016.51)

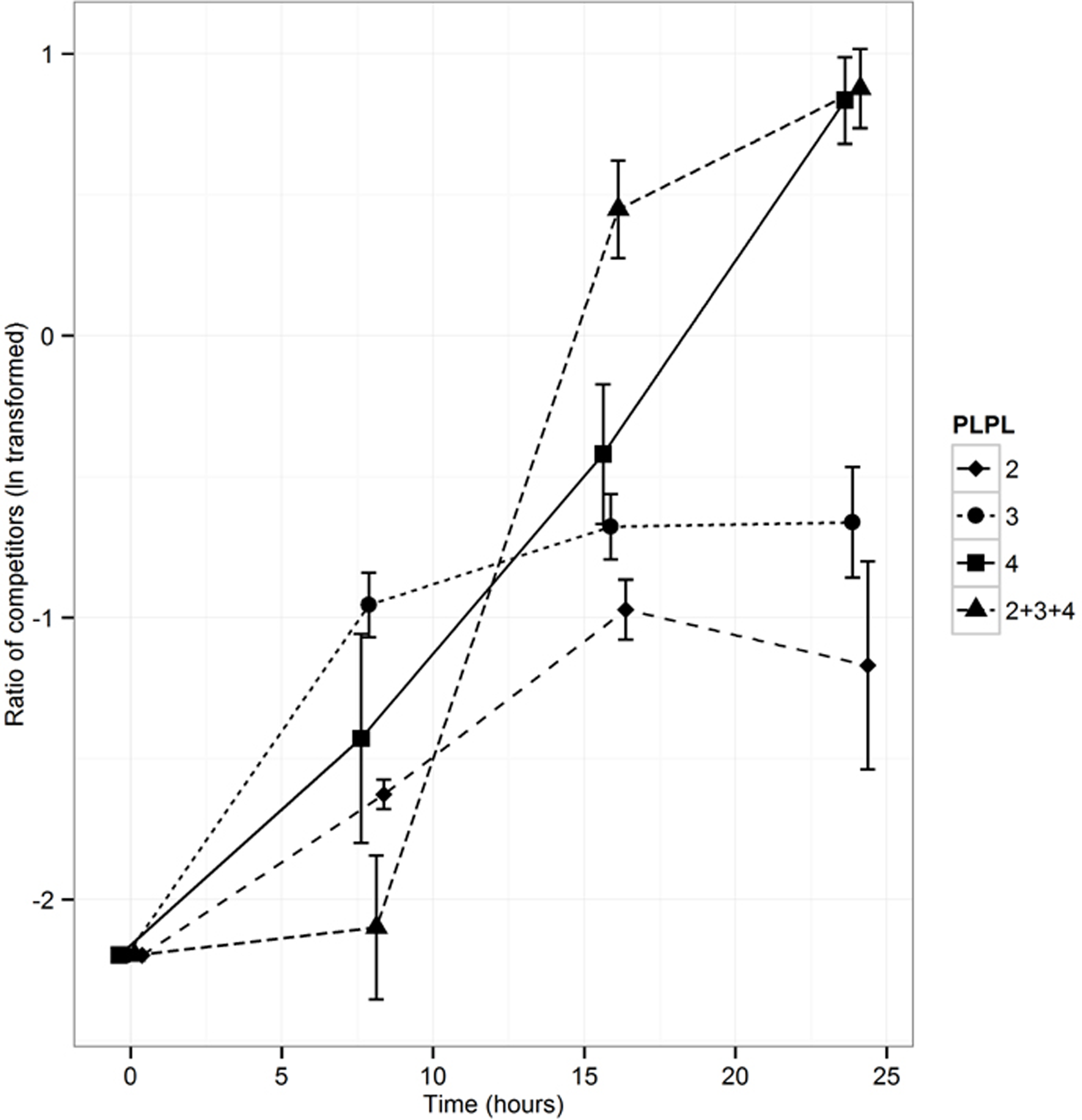

Supplement: Supplementary Figure 1 [file ismej201651x2.tif]

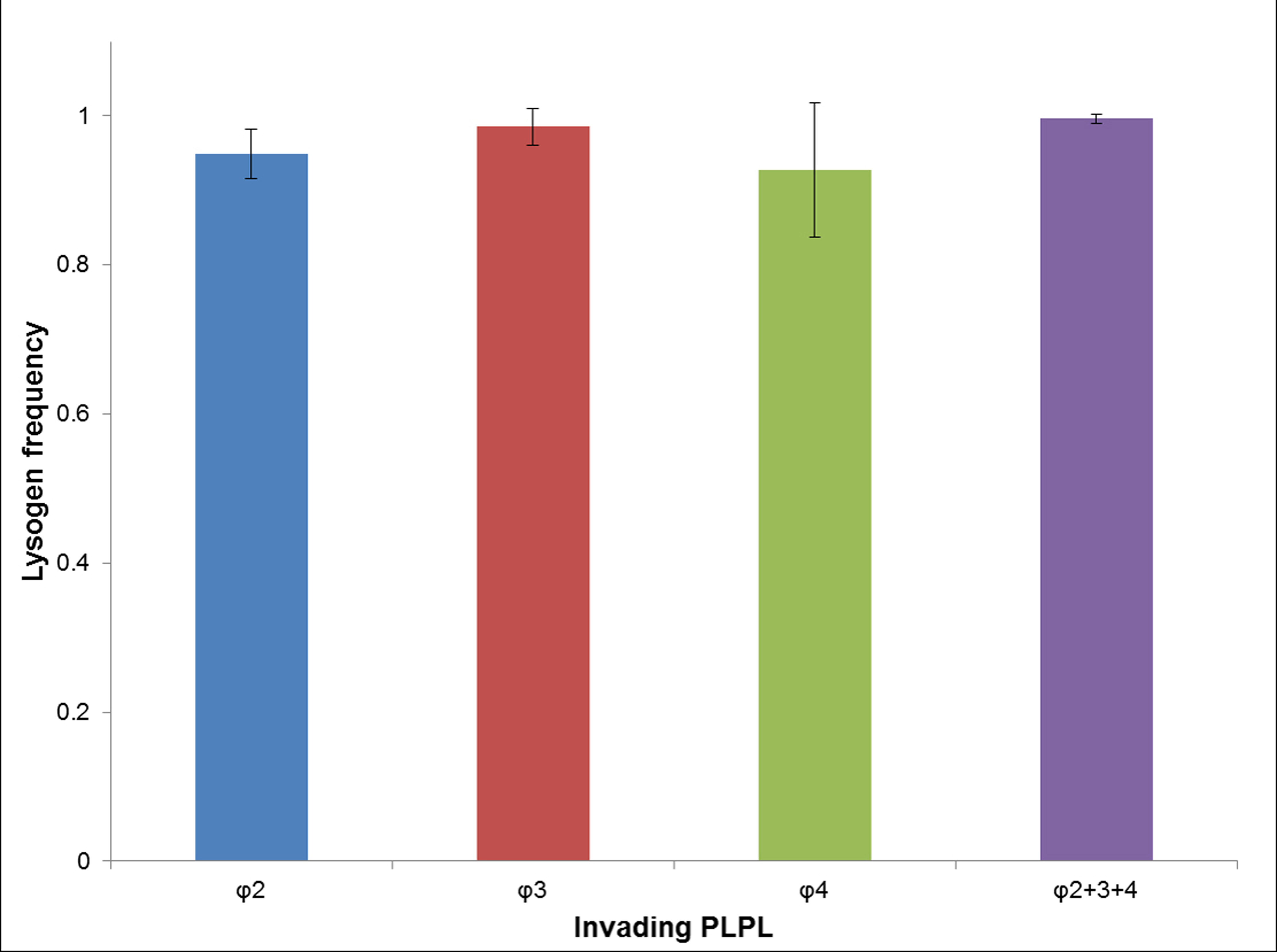

Supplement: Supplementary Figure 2 [file ismej201651x3.tif]
